# Design of new drugs for medullary thyroid carcinoma

**DOI:** 10.3389/fonc.2022.993725

**Published:** 2022-12-05

**Authors:** Yanqing Li, Ziyu Luo, Xinxing Wang, Songtao Zhang, Hu Hei, Jianwu Qin

**Affiliations:** ^1^ Department of Thyroid and Neck, The Affiliated Cancer Hospital of Zhengzhou University, Henan Cancer Hospital, Zhengzhou, China; ^2^ The Medical School of Zhengzhou University, Zhengzhou, China; ^3^ Department of Pain and Rehabilitation and Palliative Medicine, Henan Cancer Hospital, Zhengzhou, China

**Keywords:** medullary thyroid carcinoma, targeted drug, molecular mechanism, designed principle, progression

## Abstract

Medullary thyroid carcinoma (MTC) is one of the common malignant endocrine tumors, which seriously affects human health. Although surgical resection offers a potentially curative therapeutic option to some MTC patients, most patients do not benefit from it due to the difficulty to access the tumors and tumor metastasis. The survival rate of MTC patients has improved with the recent advances in the research, which has improved our understanding of the molecular mechanism underlying MTC and enabled the development and approval of novel targeted drugs. In this article, we reviewed the molecular mechanisms related to MTC progression and the principle for the design of molecular targeted drugs, and proposed some future directions for prospective studies exploring targeted drugs for MTC.

## Introduction

Medullary thyroid carcinoma (MTC) is a rare thyroid malignancy accounting for 3-5% of all thyroid malignancies (1). Thyroid cancer also includes differentiated thyroid carcinoma (DTC; including papillary, folliculous, and Hürthle cell) and anaplastic thyroid carcinoma (ATC). Different from DTC and ATC, MTC originates from the c-cells near the follicles which secrete calcitonin (2). Moreover, in comparison with DTC, MTC patients have lower morbidity and higher mortality. About 35% of MTC patients have neck metastasis and about 13% have distant metastasis (3). Similarly, the clinical manifestations of thyroid cancer are mostly neck masses. Patients with large thyroid masses may present with compression symptoms beyond the thyroid, such as dysphagia caused by the compression of esophagus, hoarseness caused by nerve compression, and dyspnea caused by the compression of the airway (4). MTC often has gene mutations, and traditional chemotherapy does not achieve satisfactory therapeutic efficacy. Therefore, the standard treatment for MTC involves surgical resection and molecular targeted therapy to improve local or systemic symptoms (5). At present, emerging targeted therapeutics have greatly alleviated the symptoms in MTC patients.

**Figure 1:**
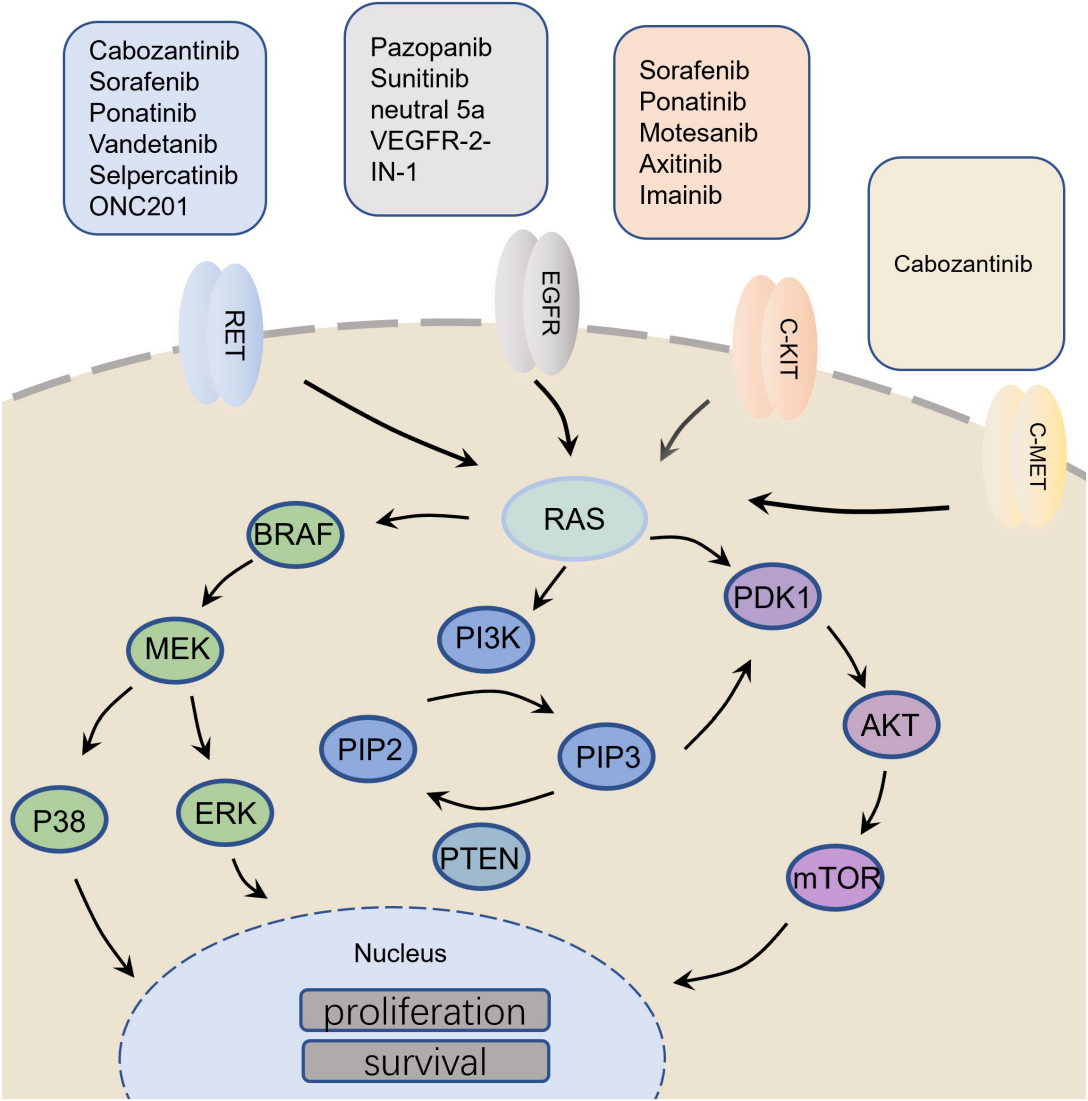
Molecular mechanism related to medullary thyroid carcinoma and its targeted drugs.

In the normal thyroid C cells, the ret proto-oncogene (*RET*), renin-angiotensin system (RAS), phosphatidylinositol-4,5-bisphosphate 3-kinase/protein kinase B/mechanistic target of rapamycin kinase *(PI3K/AKT/mTOR*), vascular endothelial growth factor (*VEGF*) and programmed death ligand-1 (*PD-L1*) are responsible for regulating the biological processes such as cell proliferation, apoptosis, differentiation, migration and immunity (6–8). However, the progression of MTC affects the normal functioning of these molecules. For example, *RET* mutation is frequently found in medullary carcinoma (9). Glial cell derived neurotrophic factor (*GDNF*) binds the GFRα receptor and the GDNF-GFRα complex binds to RET receptor resulting in the activation of tyrosine kinase domain through homo-dimerization (10). This leads to the phosphorylation of tyrosine residues in the RET protein, which mediates the activation of various intracellular signaling pathways, including *PI3K/AKT/mTOR*, *RAS/MAPK*, *VEGF*, etc. (11, 12). Moreover, in MTC, the related immune molecules, such as *PD-L1* (13), are known to mediate the inhibition of T cell activation by binding to the receptors and their ligands, which inhibits the activation and proliferation of T cells, reducing their cytotoxic effects on the tumor cells (**Figure 1**; **Table 1**). Therefore, researchers mainly focused on the above molecules while exploring anti-tumor drugs with good efficacy.

**Table 1:** Drug target and mechanism.

Tyrosine Kinase Inhibitors	Chemical-Target Interactions	Mechanism of action	PMID
Vandetanib	VEGFR2/3, EGFR (exon 19), RET (exon 7, exon 16, exon 19)	Vandetanib plays an anti-tumor role by inhibiting VEGF/MAPK and RET/RHO/JNK signaling pathways.	15604279, 17431108
Cabozantinib	VEGFR2 (Lys868, Val898, Cys 919), KIT, FLT-3, RET, MET (Met1160, Asp1222, Tyr1159, Asp1228, Phe1134)	Vandetanib inhibits MET phosphorylation and VEGF-1 induced by HGF. Cabozantinib induced phosphorylation of VEFGR2 plays an anti-tumor role.	34029957, 28411406
Selpercatinib	RET, VEGFR2	Selpercatinib highly selectively inhibits the activity of RET (rearrangement during transfection).	32557397, 32846061
Sunitinib	PDGFR, KIT VEGFR1-3 (ATP-binding domain), FLT-3, RET	Sunitinib inhibits tumor progression by inhibiting p-VEGFR-PI3K-AKT-YBX1-Snail, SMAD4/SMAD7, ERK1/2 and MAPK signaling pathways.	12646019
Sorafenib	BRAF (Trp530, Val470, Lys482, Glu500, Val503, His573, ASP593, Phe594, Phe582, Cys531), KIT, FLT-3, VEGFR (Thy1054), PDGFR (Thy857)	Sorafenib can resist tumor by inhibiting the expression of CD31, alphaSMA, pERK, VEGF, PDGF, TNFalpha, eNOS and HIF-1α/SLC7A11, BRAF/MAPK signaling pathways.	24164966
ONC201	RET, VEGFR, IGFBP2	ONC201 inhibited the transcription of RET, VEGFR2 and IGFBP2.	33536187, 3353618
Ponatinib	EGFR (Asp630, Glu520, Met524), RET, KIT	Ponatinib inhibits PI3K/Akt/mTOR, PDK1/Akt/mTOR Signaling	35318898
Motesanib	KIT (Lys623, Thy672, Cys673, Phe 811, Asp810, Glu640, His790, Cys788), VEGFR	Motesanib inhibits the activities of VEGF, PDGFR and Kit.	18596272, 20633291
Axitinib	KIT (Glu671, Cys673, Asp810, Phe811, IIE670), VEGFR (Asp1046, Phe1047, Gly1048)	Axitinib inhibits the activity of VEGF.	31046271, 25216334
VEGFR-2-IN-1	EGFR	VEGFR2-IN-1 can inhibit the proliferation and migration of cells by activating and inhibiting the expression of VEGFR2 through apoptosis.	35290929
pazopanib	VEGF	Pazopanib inhibits ER-stress, VEGF-VEGFR pathway.	28330784
lmatinib	KIT	Lmatinib inhibits the activity of KIT.	12798163

In the past decade, many novel therapeutic drug targets have been discovered and explored. Most of the anticancer drugs are known to inhibit cell proliferation, apoptosis, angiogenesis and kinase activity (14). For example, several kinase inhibitors such as imatinib, gefitinib, erlotinib, sorafenib, cetuximab and bevacizumab have been developed and approved as anticancer agents (15–17). Some of the inhibitors targeting angiogenesis and inhibitors of the *RET* pathway are still being used in the clinical practice. Vandetanib, selpercatinib, pralsetinib and cabozatinib have also been approved by the FDA for clinical use (15, 18). In addition, many other biological compounds are also currently in Phase 1-3 clinical trials (19). In this review, we discuss the molecular targets related to MTC therapy, as well as the design and challenges related to new anticancer drugs against these molecular targets.

## Mutation of *RET* gene and designing drugs targeting the *RET* gene

### Mechanism associated with *RET* gene mutation in MTC

Currently, medullary thyroid cancer is mainly thought to arise due to gene mutation. Among them, 98% of the familial cases harbor mutations in the *RET* gene, 44% cases have sporadic mutations, and 13% of the patients have mutations in the *RAS* gene (mainly *HRAS* and *KRAS*) (20). The *RET* gene is located at 10q11.2, and contains 21 exons, which encodes a Receptor Tyrosine Kinase (*RTK*). The RET protein transmits intracellular signals when growth factors bind to the receptor, thus regulating the growth, survival, differentiation and migration of neural crest-derived cells (21). *RET* mutation usually occurs in the cysteine-rich regions of the exons 10 and 11, but may also occur in the tyrosine kinase domain (22). In a few cases, papillary thyroid carcinoma has been shown to harbor rearrangements in the *RET* gene. On the contrary, MTC is characterized by point mutation in the *RET* gene. These mutations are concentrated in the exons 5, 8, 10, 11 and 13-16 (9). Among them, M918T mutation in exon 16 is the most common one in sporadic MTC, which is usually associated with the highest risk of invasive cancer (23).

### *RET* targeted drug design

Compound library screening and computer simulation are one of the main approaches for designing drugs. For example, ONC201, a small molecule anticancer agent (imipridone), is currently under phase II clinical trials for advanced thyroid cancer. ONC201 (Animidazo [1,2-a] Pyrido [4,3-d] Pyrimine derivative) was identified for the first time from NCI chemical library screening, and has a regular [3,4-e] structure. ONC201 has been reported to trigger cell death in various tumor types by inducing integrated stress response involving the transcription factor *ATF4*, transactivator *DDIT3* (*CHOP*), the pro-apoptotic protein TRAIL encoded by *TNFSF10* and the TRAIL receptor DR5 encoded by *TNFSF10B* (24). Moreover, with the recent advances in the drug design concept, computer based simulation has attracted great attention. Some studies have used the computer to generate receptor-ligand pharmacophore model for identifying small molecule inhibitors effective against the wild-type (WT) or mutant *RET* kinases. Following this, small molecule inhibitors consistent with the model were selected from the natural product database. Then, the obtained drugs were subjected to molecular docking with the *RET* WT kinase domain, and drugs with better *RET* binding free energy scores were screened out (25). For example, Hit1 (ZINC02123418), Hit2 (ZINC02113839) and Hit3 (ZINC04030012) were small molecule inhibitors that were identified as the hits against WT *RET*, and V804M and V804 *RET*. However, the potency of the above drugs has not yet been tested *in vitro* and *in vivo*.

**Figure 2:**
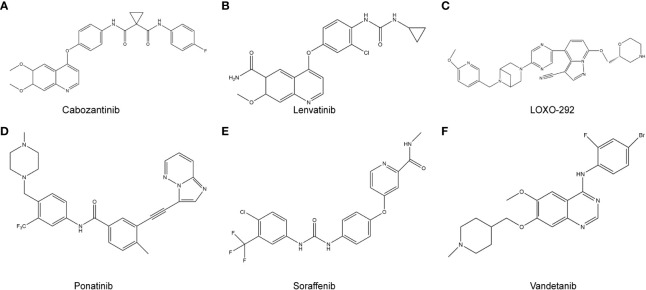
RET target-related chemotherapy drugs. **(A)**, cabozantinib; **(B)**, Lenvatinib; **(C)**, LOXO-292; **(D)**, Ponatinib; **(E)**, Sorafenib; **(F)**, Vandetanib.

ATP competitive kinase inhibitors have attracted much attention due to their high affinity and clear action sites, and are also the most studied type of kinase inhibitors. ATP competitive kinase inhibitors compete with the substrate ATP to bind to the ATP binding pocket of the kinase, thereby inhibiting the kinase activity in the signaling pathway. According to the ligand protein binding pattern, ATP competitive kinase inhibitors are generally classified into type I and type II (26). Type I inhibitors bind to active kinases with a “DFG (Asp-Phe-Gly) - in” conformation, while type II inhibitors bind to inactive kinases with a “DFG - out” conformation. Type II inhibitors have been reported to have better cellular potency and kinase selectivity than the type I inhibitors. Because the type II inhibitors occupy the non-conservative hydrophobic pockets produced by DFG flip, they have an expanded chemical space for drug molecule discovery (27). Finally, type II inhibitors show lower affinity for ATP than the for the active conformation of the *RET* kinase. Common type II *RET* inhibitors against *RET*-positive cancers include cabozantinib, sorafenib, ponatinib etc., all of which show good efficacy (**Figure 2**). Additionally, FDA has also approved multi-kinase inhibitors targeting *RET* kinase such as vandetinib (inhibits multiple receptors: *RET*, *EGFR* and *VEGF2*) and cabozantinib (inhibits *RET*, *C-Met*, *VEGFR2* and *AXL*), for the treatment of MTC (28, 29). The above drugs were efficacious in MTC patients, especially those harbouring the RET M918T mutation, according to ZETA and EXAM trials. However, the “functional acquisition” of mutation in the *RET* gate-keeper residue Val804, confers resistance to most of the known *RET* inhibitors, such as vandetanib (15). There is still controversy about the correlation between *RET ^V804^* mutation and vandetanib resistance. Carlomagno F et al. proved *in vitro* that V804 mutation caused cell resistance to vandetanib (30). They explained that V804 is the gatekeeper in ATP-binding pocket. Its mutation affects the stability of protein, thus endowing it with resistance to vandetanib. However, Samuel A et al. found no correlation between V804 mutation and vandetanib (31). They think that in their experiment, there are a large number of patients with unknown *RET* mutation status, because their paraffin blocks or slides don’t have enough quantity or quality of DNA for complete analysis. The small number of *RET* negative patients means that subgroup analysis of PFS and objective remission rate according to *RET* mutation status is uncertain. Therefore, the difference of curative effect of vandetanib on *RET ^V804^* mutation patients needs to be included in more patients for further analysis. To overcome this drug resistance, researchers developed selpercatinib (LOXO-292) and pralsetinib (BLU-667) against the mutant forms of the *RET* gate-keeper-V804M/L (32). Selpercatinib and pralsetinib were designed not only to target the gatekeeper mutations (such as V804) but also to minimize the serious adverse events recorded with multi TKIs. This was achieved due to the higher affinity of these drugs for the RET receptor. It is worth mentioning that even *RET* selective inhibitors that overcome the gatekeeper mutation are unable to target the acquired solvent front mutation (*RET ^G810S/R/C^*). TPX-0046, a new drug, has shown promising results in inhibiting the solvent frontier mutation G810R (33).

## The *RAS* signaling pathway and designing targeted drugs against this pathway

### The role of *RAS* signaling pathway in MTC

About 70% of MTC cases without recognizable *RET* mutation are attributed to mutations in the *RAS* oncogene. The *RAS* gene encodes GTP enzymes, which regulates a variety of downstream pathways including the *MEK/ERK* and *PI3K/AKT* pathways, and plays important roles in a variety of processes including cell growth and proliferation, apoptosis and differentiation (34). About 20% of cancers harbor activating mutations in the *RAS* oncogene. Activating mutation in *HRAS* is the most common in MTC, and the proportion of *KRAS* and rare *NRAS* mutations is low (35). *RAS* and *RET* mutations are usually mutually exclusive in MTC, except for a few anecdotal reports of mutations in both genes (36). As with other *RAS*-driven cancers, mutations in MTC mainly occur in the exons 2 and 3. The most common activating mutations in *RAS* occur in the G12, G13 and Q61 residues (37).

### Design of *RAS* inhibitors

For several decades since the discovery of *RAS*, no drugs were developed that directly targeted *RAS* and inhibited its abnormal function (38). It was not until May 2021 that the FDA approved the first *KRAS* inhibitor Sotorasib for clinical use, which opened the doors for the clinical application of *KRAS* inhibitors. There are several reasons that hamper the development of drugs that directly target the *RAS* oncogene. Firstly, the RAS protein has a strong binding affinity to GTP, but the concentration of GTP in normal cells is high, therefore, it is difficult to develop competitive inhibitors that can directly target GTP. Secondly, the surface of the RAS protein is similar to a smooth sphere, lacking the pocket for binding to drugs (39). Finally, *RAS* is an essential protein that regulates normal cellular activity besides having oncogenic role in different types of tumors (40). Targeted small molecule inhibitors not only inhibit the activity of mutant *RAS*, but also inhibit the activity of the WT *RAS*, thus causing toxic side effects and adverse reactions in cancer patients. Therefore, it is difficult to develop competitive inhibitors that directly target *RAS*. However, with the analysis of the crystal structure of *RAS*, researchers found that there were two switches on its surface, namely the switch I and switch II. Among them, the switch II pocket in the protein showed high variability (41). Switch II induced conformational change of the protein in the switch II region, which inactivated *RAS* and blocked signal transmission through the *RAS* pathway. The small molecule *RAS* inhibitor sotorasib, bound to the switch II pocket and only engaged with the inactive GDP state of *KRAS ^G12C^*. Sotorasib was shown to promote therapeutic effects in lung cancer (42), however, similar studies have not yet been conducted in MTC patients to evaluate the therapeutic effects of sorafenib in MTC.

**Figure 3:**
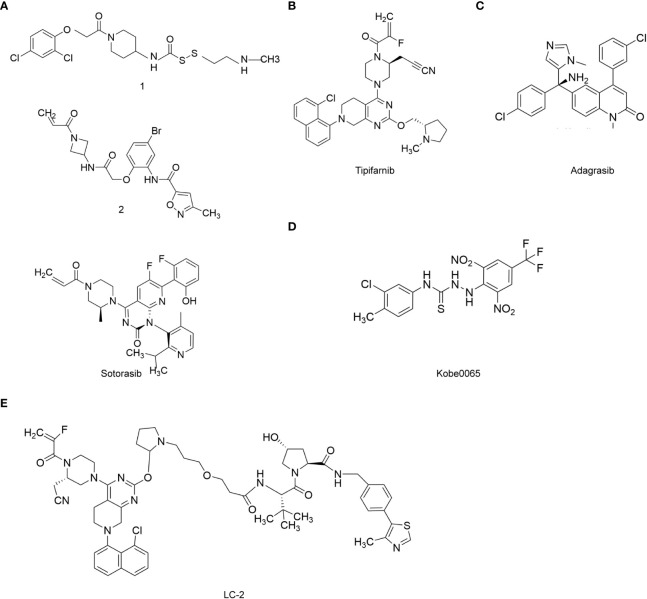
RAS target-related chemotherapy drugs. **(A)**, Sotorasib; **(B)**, Tipifarnib; **(C)**, Adagrasib; **(D)**, Kobe0065; **(E)**, PROTAC LC-2.

Amgen developed sotorasib using a fragment-based approach for their drug design. After analyzing the crystal structure of *RAS* with X-rays or magnetic resonance imaging, the researchers used molecular simulation and covalent binding methods to find allosteric binding sites in the target protein. The designed drug covalently bound to the Cys 12 site, leaving *KRAS* in an irreversible inactive conformation. To achieve this goal, they first screened 380 disulfide compounds with weak electrophilic properties and identified compound 1 (structural formula is shown in **Figure 3A**) as the specific compound with a strong binding affinity to the KRAS-GDP complex (43). The eutectic structure showed that compound 1 bound to *KRAS^G12C^* at the switch II region, and the disulfide bond of compound 1 was substituted and bound to the mutant Cys 12 residue. Researchers then replaced the disulfide structure with acrylamide, which was more electrophilic to enhance the binding strength to the Cys 12 residue. It is necessary to couple the compound containing the reactant group and the drug skeleton to generate a single molecule. Therefore, Amgen used the Chemotype Evolution platform of Carmot Therapeutics to identify such compounds. Compound 2 (structural formula is shown in **Figure 3A**) was identified by rapid screening and analyzed by several biochemical assays and mass spectrometry, and after a series of skeleton fusions, structural optimization, and rigorous testing of its *in vitro* and *in vivo* potency, sotorasib was finally developed (44).

Adagrasib (MRTX849) was developed as a drug with a similar mechanism of action as sotorasib. Adagrasib also inhibited *KRAS^G12C^* and was developed by Mirati Therapeutics. Adagrasib showed good efficacy in clinical trials against patients with advanced/metastatic NSCLC harboring *KRAS^G12C^* mutation. Adagrasib monotherapy showed an objective response rate (ORR) of 45% and a disease control rate of 96% (45). Therefore, it may be a potential drug for MTC patients with *KRAS* mutation. The molecular formula of Adagrasib is C32H35ClFN7O2, the relative molecular weight is 604.12, and its structural formula is shown in **Figure 3C**. The design concept and synthetic process of adagrasib are similar to that of sotorasib.

*HRAS* mutation is also a common type of mutation in MTC patients, however, there are relatively few studies reporting targeted drugs for *HRAS* as compared with *KRAS*. Among them, tipifarnib is an FTase inhibitor showed good efficacy in patients with *HRAS* mutations (46). The molecular formula of tipifarnib is C27H22Cl2N4O and the molecular weight is 489.40. The structural formula of tipifarnib is shown in **Figure 3B**.

It is worth mentioning that in the conventional synthesis process of tipifarnib and active R-configuration drugs involves obtaining active pharmaceutical ingredients through chiral resolution, which results in a large amount of wastage of intermediates. The intermediates can be racemized by using appropriate acidation during the synthetic process, thus improving the synthesis efficiency of the drug. Tipifarnib was first developed by Johnson & Johnson and submitted to the FDA for new drug application in 2004. In April 2021, tipifarnib was approved by the FDA for the treatment of head and neck squamous cell carcinoma patients who failed to respond to platinum based chemotherapy and had *HRAS* mutation ≥20% (47). Also, Kobe0065, is also a small molecule drug targeting *HRAS*, which has not yet been used for treating MTC patients in the clinic. This drug holds a great promise for clinical use in MTC patients. It is known to inhibit the interaction between HRAS and cRaf1 (48), and effectively prevents the binding of HRAS-GTP to cRaf1-RBD. The molecular formula of Kobe0065 is C15H11ClF3N5O4S, and the molecular weight is 449.79. The structural formula of Kobe0065 is shown in **Figure 3D**.

All targeted drugs face the problem of drug resistance, and drugs targeting *RAS* are no exception. Degradation of chimeras with targeted proteins is a solution to overcome drug resistance, for example, PROTAC LC-2 (structural formula shown in **Figure 3E**), was developed by CREW et al., which was the first drug that degraded endogenous *KRAS^G12C^* mutants (49). The precursor of LC-2 was LC-1, which contained the adagrasib molecule, the E3 ubiquitin ligand and the ligand for both of them. Due to the presence of hydrolyzable amides in LC-1, KRAS could not be degraded effectively. Thus, LC-2 was optimized to solve this problem and its ability to degrade KRAS was greatly enhanced. Firstly, the adagrasib fragment in LC-2 covalently bound to *KRAS^G12C^*, then the E3 ubiquitin ligase protein was recruited to degrade the *KRAS^G12C^* mutant. PROTAC drugs are independent of the active site and do not require continuous binding to the target. Although it has not been applied to MTC patients, PROTACs may be an effective strategy for treating MTC patients and to overcome the problem of *RAS* inhibitor resistance.

## The *PI3K/AKT mTOR* signaling pathway and designing drugs against this pathway

### The role of *PI3K/AKT/mTOR* signaling pathway in MTC

The success of *RET* inhibition in MTC therapy suggests that the downstream signaling pathway activated by *RET* may also be an effective therapeutic target. It was found that the proteins in the PI3K/AKT/mTOR pathway were preferentially activated in hereditary *RET* positive cases (50). PI3K is activated after its phosphorylation and AKT is activated by phosphorylation on the cell membrane. *AKT* regulates downstream processes including the inhibition of *p27*, translocation of FOXO in the cytoplasm, activation of *PtdIns-3ps*, mediating the effects of *p70* or *4EBP1* on transcriptional regulation and activation of the *mTOR* signaling pathway. The *mTOR* gene is located on chromosome 1p36.22 and contains 60 exons. *mTOR* activation is an early event in the transformation of C cells, which plays a role in the early stage of MTC tumorigenesis. *mTOR* encodes serine/threonine kinase belonging to the phosphatidylinositol kinase-related kinase family, which is involved in the regulation of cell proliferation, apoptosis, cell cycle, angiogenesis, metabolism and protein synthesis.

### Design of *PI3K/AKT/mTOR* inhibitors

**Figure 4:**
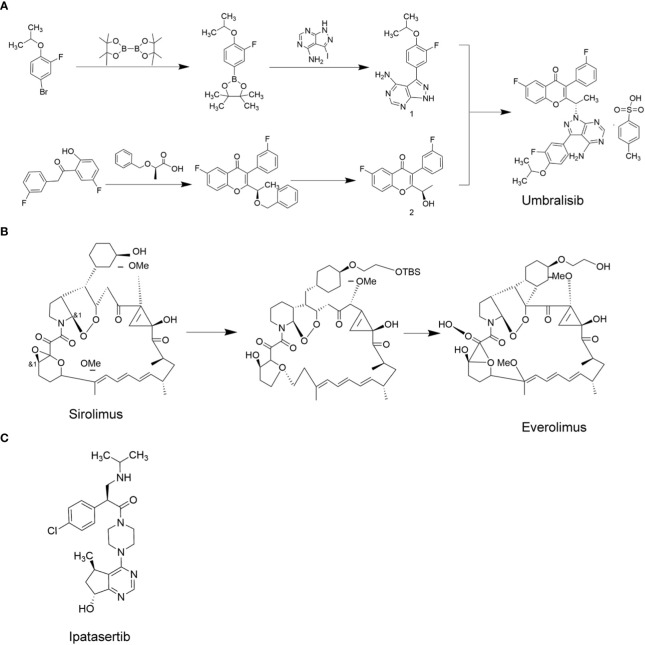
PI3K/AKT/mTOR target-related chemotherapy drugs. **(A)**, Umbralisib; **(B)**, Everolimus; **(C)**, Ipatasertib.

*PI3K* inhibitors can be divided into three categories. The first category consists of broad spectrum *PI3K* inhibitors, such as buparlisib (BKM120). In a clinical trial on thyroid cancer patients, buparlisib showed good therapeutic effects, but 63% of the patients developed serious adverse reactions (51). The second type is *PI3K* subtype selective inhibitors, such as *PI3Kα* selective inhibitor piqray (Alpelisib) and *PI3Kδ* selective inhibitor ukoniq (Umbralisib). So far, the efficacy of these drugs have not been tested in MTC patients. The third category consists of *PI3K* and *mTOR* dual-target inhibitors, such as dactolisib (BEZ235) (52). Dactolisib was reported to significantly inhibit the proliferation of thyroid cancer cells *in vitro* and in mice (53). However, it has not been studied in MTC patients. The clinical use of broad-spectrum *PI3K* inhibitors is limited due to their severe adverse effects. In contrast, the *PI3K* subtype selective inhibitors have greatly reduced the incidence of adverse events and have shown good therapeutic efficacy. Umbralisib (54) is a selective *PI3Kδ* inhibitor with a molecular formula of C38H32F3N5O6S, and molecular weight of 743.75, and its synthetic route is depicted in **Figure 4A**. In view of its excellent therapeutic effect in relapsed/refractory B-cell non-Hodgkin lymphoma and chronic lymphocytic leukemia, umbralisib may be a potential new therapeutic drug for MTC (55). **Table 2** shows the list of *PI3K/AKT/mTOR* pathway inhibitors that are currently under investigation.

**Table 2:** Specific targets of Protein Tyrosine Kinase/RTK drugs acting on VEGFR.

	Vandetanib	Sorafenib	Sunitinib	Ponatinib		Pazopanib	PD173074	VEGFR2-IN-1	VEGFR-2-IN-10
Target (IC50)	VEGFR2 (40 nM)	VEGFR3 (20 nM)	VEGFR2 (80 nM)	VEGFR2 (1.5 nM)		VEGFR1 (10 nM)	FGFR1 (25 nM)	VEGFR2 (19.8 nM)	VEGFR2 (0.7 μM)	
	VEGFR3 (110 nM)	Braf (22 nM)	PDGFRβ (2 nM)	PDGFRα (1.1 nM)		VEGFR2 (30 nM)	VEGFR2 (100 nM)			
	EGFR/HER1 (500 nM)	Raf-1 (6 nM)		FGFR1 (2.2 nM)		VEGFR3 (47 nM)				
		VEGFR2 (90 nM)		c-Kit (12.5 nM)		PDGFRβ (84 nM)				
		PDGFRβ (57 nM)				FGFR1 (140 nM)				
		Braf^V599E^ (38 nM)				c-Kit (74 nM)				
		c-Kit (68 nM)			c-Fms (146 nM)					

*AKT* is an effector in the *PI3K/AKT/mTOR* pathway, and is a promising target for cancer treatment. The *AKT* kinase family includes *AKT1*, *AKT2*, and *AKT3* (56). *AKT* inhibitors are classified into three categories based on how they inhibit the activity of AKT. ATP competitive inhibitors are the first category of *AKT* inhibitors that suppress AKT phosphorylation by competing with ATP. The second category consists of allosteric inhibitors, which inhibit the interaction between *AKT* and its substrate through different conformations. Lastly, the PH domain inhibitors inhibit AKT by competing with PIP3 for the binding sites on the PH domain, preventing PIP3 induced migration of AKT to the cell membrane (57). ATP competitive inhibitors are currently the focus of research for *AKT* inhibitors. Some of the related small molecule drugs include ipatasertib, uprosertib, etc. The allosteric inhibitor (MK-2206) has been reported to show stronger *AKT* inhibition in the laboratory than the ATP competitive inhibitors, but the clinical data for MK-2206 is not yet available. The PH domain inhibitor perifosine, did not show satisfactory results in clinical trials. A phase III randomized, double-blind, placebo-controlled trial of perifosine for rectal cancer showed no overall survival benefit (58). However, *in vitro* experiments showed that perifosine could effectively inhibit the growth of thyroid cancer cells (59). However, it has not yet been tested in MTC patients. Among all the *AKT* inhibitors, ipatasertib (structural formula shown in **Figure 4C**) is the most advanced compound in clinical research. Due to the unique chiral pyrimidine cyclopentanol structure of ipatasertib, its inhibitory effect on AKT was found to be 620 times that of its homologous protein PKA, and its IC50 values against AKT1, AKT2 and AKT3 in *in vitro* assays were 5, 18 and 8nmol/L, respectively (60). Ipatasertib has not yet been tested in MTC patients, which may be a new direction for treating MTC. Ipatasertib was developed by Array BioPharma, and currently it is promoted by Roche.

The *mTOR* kinase family includes *mTOR1*, *mTOR2* and *mTOR3*, of which *mTOR1* and *mTOR2* are associated with tumorigenesis (61). *mTOR1* is a downstream effector of several common signaling pathways, including the *PI3K/AKT* and *MAPK* signaling pathways (62). *mTOR* is overactive in a variety of cancers, making it an ideal target for cancer therapy (63). mTOR inhibitors can be divided into two types. The first category consists of rapamycin and its analogs, and the second category consists of ATP-competitive inhibitors (64). The former inhibits *mTORC1* and the latter inhibits *mTORC1/2*. Rapamycin has not been approved for cancer therapy due to its immunosuppressive effects. However, its analogue, everolimus, was reported to promote relatively low immunosuppressive effects and has been approved by the FDA for treating renal cell carcinoma and pancreatic derived neuroendocrine tumors, advanced hormone receptor-positive *HER-2* negative breast cancer, gastrointestinal neuroendocrine tumor, and giant cell astrocytoma (65, 66). In addition, in MTC patients, everolimus was reported to induce significant anti-tumor effects (67). Everolimus is currently the most widely used *mTOR* inhibitor. The synthesis of everolimus uses sirolimus as raw material, and everolimus precursor is obtained through etherification reaction with the sulfonate side chain. The everolimus precursor is then processed by desilication ether to obtain everolimus. **Figure 4B** depicts the synthetic process for everolimus.

Moreover, broad-spectrum *PI3K* and *mTOR* dual-target inhibitors are also the major focus of cancer research, but currently, drugs belonging to this category have not yet been approved by the FDA for clinical use. For example, it was found that dactolisib (BEZ235) inhibited the growth of thyroid cancer through *p53*-dependent/independent *p21* up-regulation (53). However, the development of *PI3K* and *mTOR* dual-target inhibitors is an effective strategy to overcome resistance to *PI3K* or *mTOR* single-target inhibitors. On the basis of the existing structures of the dual-target inhibitor compounds, different series of compounds can be designed and synthesized by computer simulation, using skeleton transition strategies. Through the study of structure-activity relationship and the effect of the drugs on *in vitro* proliferation of cancer cells, dual-target inhibitors with good activity and low toxicity can be selected for broad therapeutic applications.

## Angiogenesis and drugs targeting the angiogenesis pathway

### The role of angiogenesis in MTC

Angiogenesis and lymphangiogenesis are indispensable for tumor occurrence and metastasis. Angiogenesis and lymphangiogenesis are distinct cellular processes. Angiogenesis refers to the re-growth of capillaries from existing blood vessels, while lymphangiogenesis refers to the development of new lymphatic vessels. The VEGFR2 receptor tyrosine kinase is expressed by vascular endothelial cells and is activated by VEGF-A produced by the immune cells in the tumor and the tumor microenvironment (TME) (68). *VEGFR1*, *-2*, *-3*, *VEGF-A* and *VEGF-C* have been reported to be highly expressed in MTC patients. Moreover, the overexpression of *VEGFR2* is related to the prognosis of metastatic MTC patients (14). *VEGF-A* also regulates lymphangiogenesis through the participation of *VEGFR3*. *VEGF-C* and *VEGF-D*, produced by several immune cells in the TME, are known to stimulate lymphangiogenesis and metastasis of tumors through the combination of *VEGFR3* and lymphatic endothelial cells. *EGFR* is a cell surface protein belonging to the *ErbB* receptor family, and about 30% of epithelial cancers have dysregulated *EGFR* signaling, amplification or mutation. *EGFR* is involved in cell growth stimulation and signal transduction, as well as in the activation of RET kinase, which is related to thyroid cancer progression and invasion (69).

### Drug design for targeting angiogenesis in MTC

Many kinds of targeted drugs have been developed for MTC in the past 20 years. Tyrosine kinase receptors are involved in cancer cell proliferation, angiogenesis and lymphangiogenesis. Several tyrosine kinase inhibitors (TKIs) have been developed as new drugs for the treatment of MTC, which were successful in suppressing disease progression. At present, FDA has approved vandetanib and cabozatinib for MTC treatment. Vandetanib was synthesized by adding a basic side chain at the C-7 position of quinazoline nucleus of 4- anilino quinazoline compounds (70). The study found that early use of vandetanib could improve the prognosis of MTC patients (71). The chemical structural formula of Cabozatinib is 1-n-[4-(6,7-dimethylquinolin-4-yl) oxyphenyl]-1-n’-(4-fluorophenyl) cyclopropane-1,1-dicarboxyamide, and it has obtained 7579473 patents (WO 2005030140 A2) in the United States (72). In a study on 330 patients with metastatic MTC, it was found that 140 mg/d Cabozantinib could significantly improve the PFS of patients (73). Previously, studies have reported that targeting the C-4’ position of *VEGF-R2* and *PDGF-Rβ* alters the inhibitory properties of drugs against *VEGF-R2* and *PDGF-Rβ*. For example, neutral 5a is an effective inhibitor of *VEGF-R2*, while acidic 5b is an effective inhibitor of *PDGF-Rβ*. In order to improve the solubility of indoline -2- one and broaden its kinase inhibition spectrum, researchers introduced various basic side chains at the C-4’ position of 5a. As a kind of indoline -2- one drug, sunitinib is a multi-target tyrosine kinase inhibitor. Phase II clinical trials showed that sunitinib had obvious curative effects in patients with advanced, locally advanced or metastatic medullary thyroid cancer (74). Also, ponatinib was reported to effectively inhibit *Abl*, *PDGFRα*, *VEGFR2*, *FGFR1* and Src, thus suppressing metastasis in MTC patients. Ponatinib mainly formed five hydrogen bonds with the target protein, one skeleton with M318 in the hinge region, one skeleton with D381, one side chain with E286, and two from methylpiperazine groups (75, 76).

In recent years, few novel targeted drugs against *VEGRF* have been reported. In 2021, Cho et al. synthesized VEGFR-2-IN-10 by directly promoting the interaction between the indole core of voacangine (Voa) and the active residues of the kinase domain of *VEGFR2* (Leu840, Val848, Ala866 and Leu1035) through hydrophobic interaction. Firstly, VEGFR-2-IN-10 showed no cytotoxicity. Secondly, it had a strong inhibitory effect on *VEGF* mediated angiogenesis. This was mainly because two oxygen atoms of Voa formed hydrogen bonds with Asn923 and Cys919 in the hydrophobic pockets promoting higher affinity and direct interaction with *VEGFR2*. In order to replicate the interaction between Voa and *VEGFR2*, researchers designed indole core (5- methoxy -1H- indole) and various functional groups that were different from cycloheptane (77). In 2022, Nafie and Boraei et al. conducted the reaction between 4- amino -5-(1H- indole -2- yl) -2,4-dihydro-3h-1,2,4-triazole -3- thione 1 and indole -3- formaldehyde 2, pyridine -2- formaldehyde 3, pyridine -3- formaldehyde 4 and pyridine-1, respectively, leading to the synthesis of the drug VEGFR2-IN-1. VEGFR2-IN-1 inhibited the proliferation and migration of cancer cells by activating apoptosis and inhibiting the expression of *VEGFR2* (78).

## Immunotherapy and targeted drug design

### Immune mechanisms associated with MTC

**Figure 5:**
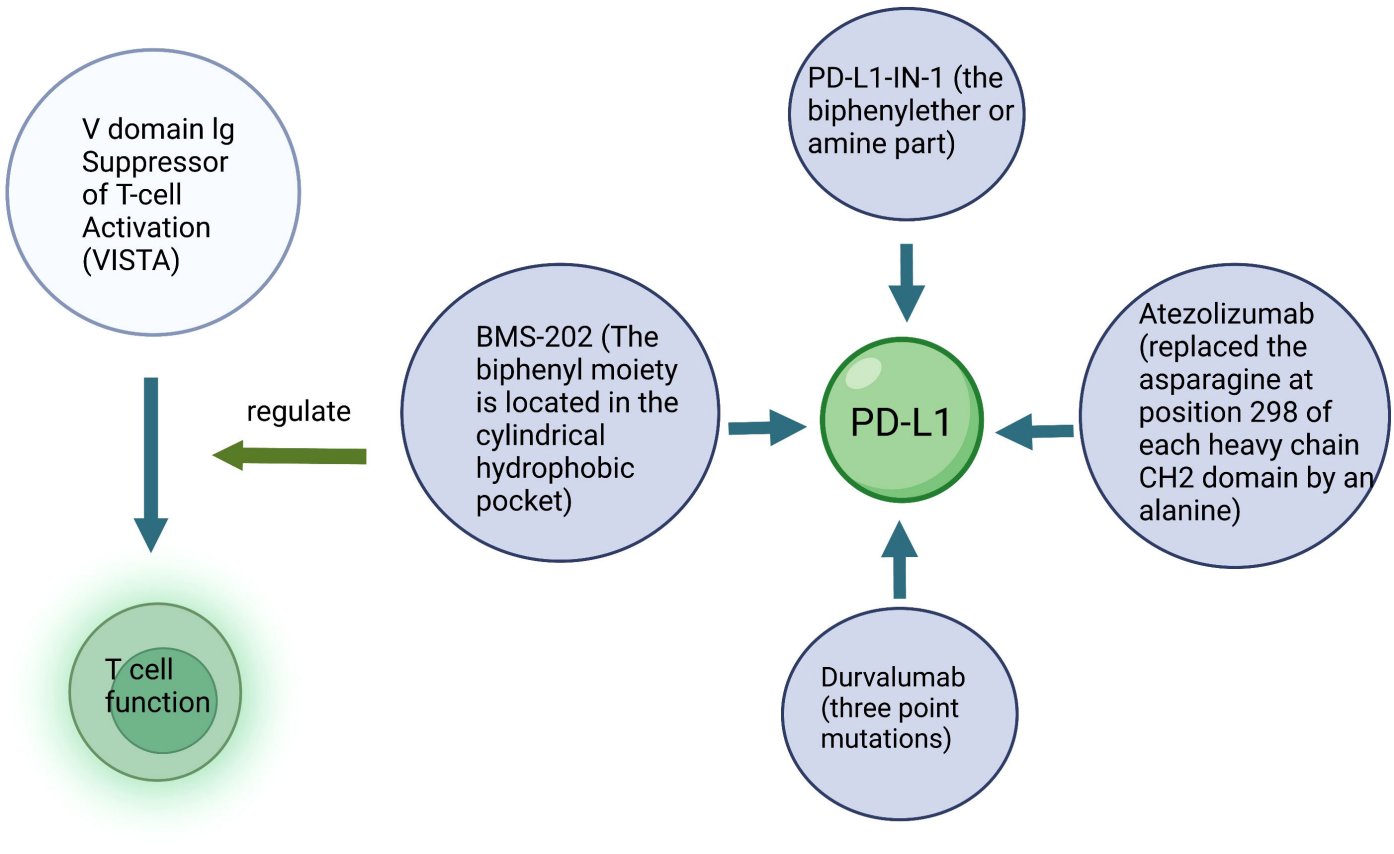
Schematic diagram of immunotherapy. Key structure of a specific drug and the target cells are described.

The TME in TC is rich in immune cells, therefore immunotherapy is a potential strategy for treating TC (79). Immunotherapy enhances antitumor immunity in patients, and through its inherent specificity, exerts cytotoxic effects on cancer cells with minimal side effects (80). Immunotherapy mediates its effects mainly by increasing the tumor specific immune response, and by suppressing the immunosuppressive nature of the tumor (**Figure 5**). For example, the dendritic cell (DC) vaccine with heterologous calcitonin polypeptide (in mice) and allogeneic tumor cell lysate (in humans) has been demonstrated to increase the tumor specific immunity in MTC patients (81). Moreover, some stromal cells in the TME are known to induce immune dysfunction, such as inhibiting DC differentiation and maturation, and further inducing CD4+ T cells to differentiate into immunosuppressive regulatory T cells (Treg). Treg express the cytotoxic T-lymphocyte antigen-4 (*CTLA-4*) and programmed death receptor-1 (*PD-1*), which are known to inhibit antitumor immune response. These inhibitory molecules were identified as “immune checkpoints” (82). *CTLA-4*, also known as *CD152*, is expressed by activated CD4 + and CD8+ T cells. *CTLA-4* is a member of the immunoglobulin superfamily, which inhibits T cell activation after binding with its ligand B7. Therefore, blocking *CTLA-4* stimulates the activation and proliferation of immune cells, inducing or enhancing the antitumor immune response. In addition, T cell immunotherapy using chimeric antigen receptor and macrophage targeting therapy have shown significant therapeutic efficacy in MTC patients (83).

*PD-1*, a member of the *CD28* superfamily, is expressed by activated T cells, B cells and myeloid cells. It has two ligands, namely, *PD-L1* and *PD-L2*. The combination of *PD-1* and *PD-L1* mediates the inhibitory signal of T cell activation, which inhibits the activation and proliferation of T cells and plays a negative regulatory role similar to that of *CTLA-4*. *PD-L1* expression has also been reported as one of the important factors associated with T cell failure during chronic viral infection and cancer. Therefore, blocking *PD-1/PD-L1* helps to rebuild T cell function, which is of great significance for tumor growth inhibition. Anti-*PD-1/PD-L1* inhibitors have been reported to enhance the immune response in certain patients with malignant tumors such as melanoma, lung cancer and renal cell carcinoma.

In most cases, tumors evade the surveillance of immune system through many mechanisms, including the down-regulation of MHC molecules, suppressing immune response and inducing tolerance to tumor antigens (84). However, little is known about the immune escape mechanism of MTC (85). Mitsiades et al. reported a potential role of *FasL* in immune evasion and the progression of thyroid cancer (86). FasL is a transmembrane protein belonging to the tumor necrosis factor (*TNF*) family, which induces apoptosis by binding and activating the Fas (APO-1/CD95) receptor. *Fas/FasL* system plays an important role in immune homeostasis and participates in T cell-mediated cytotoxicity. *FasL* has been reported to be expressed in five thyroid cancer cell lines and induces apoptosis of lymphocytes expressing *FasL*. Although its expression is low or absent in MTC, the above studies indicate a potential role of *FasL* in the progression and immune evasion of thyroid cancer.

### Designing drugs for cancer immunotherapy

Because little is known about the immune escape process of MTC, the current focus of drug development is to identify the molecular pathways leading to the formation of new antigens, so as to initiate the immune response. Such new antigens may serve as effective therapeutic targets. Currently, most of the drugs target *PD-L1*. In order to reduce the antibody-dependent cytotoxicity (ADCC) of traditional anti-*PD-L1* agents, researchers designed three classic immunotherapeutic agents for MTC, namely durvalumab, atezolizumab and nivolumab (87). Durvalumab is a selective, high-affinity human IgG1 mAb designed to prevent ADCC (88). Stewart et al. altered the constant domain of durvalumab by three point mutations, and reduced the likelihood of triggering effector functions that could lead to non-specific cytotoxic effects on healthy cells expressing *PD-L1* (89). Atezolizumab is a fully humanized IgG1mAb designed to interfere with the binding of PD-L1 ligands to their two receptors, PD-1 and B7.1. Deng et al. replaced the asparagine at position 298 of each heavy chain CH2 domain by an alanine (90). This FcgR-binding-deficient structure meant that atezolizumab could not bind to the Fc receptors on phagocytes, thereby suppressing ADCC. Wang et al. found the desired drug from a transfected CHO cell line by sequencing the variable region and grafting it onto human kappa and *IgG4* constant region sequences containing the *S228P* mutation (91).

**Figure 6:**
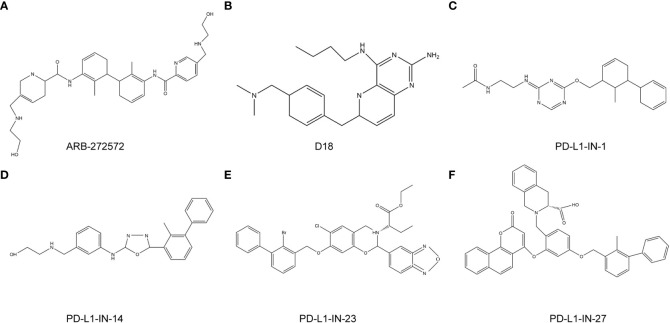
anti-PD-L1 targeted chemotherapy drugs. **(A)**, ARB-272572; **(B)**, D18; **(C)**, PD-L1-IN-1; **(D)**, PD-L1-IN-14; **(E)**, PD-L1-IN-23; **(F)**, PD-L1-IN-27.

Small molecule inhibitors targeting *PD-L1* are still being explored (**Figure 6**). PD-1/PD-L1-IN-23 (BMS-202) is an ester prodrug of L7. The biphenyl moiety of BMS-202 is located in the cylindrical hydrophobic pocket of the PD-L1 dimer protein to block the PD-1/PD-L1 interaction. In addition, combination of the benzoxadiazole compound L1 and BMS-1016 had a good arrangement pattern. The slip docking fractions of BMS-1016 and L1 were 13.085 and 13.195 kcal/mol, respectively, which indicated that L1 could be a new lead molecule for future *PD-L1* inhibitor design (92). The combination of PD-L1-IN-1 and PD-L1 proteins enhances the antitumor immune activity of cells. PD-L1-IN-1 was shown to significantly increase the release of interferon-γ and induce apoptosis in cancer cells, and induce low cytotoxicity towards healthy cells. Researchers speculated that the biphenyl ether or amine part of the compound was the driving group that bound to the surface of PD-L1. As a result, they retained this key model, replaced the polar chain with a small number of polar groups, designed some triazines7-21, and obtained patent approval (BMS-202) (93). Another study showed that PD-1/PD-L1-IN-14 (compound 17) inhibited PD-1/PD-L1 interaction, and promoted the dimerization and degradation of PD-L1 (94). In addition, PD-1/PD-L1-IN-27 had the ability to activate CD8+T cells and reduce T cell depletion. Targeting PD-1/PD-L1-IN-27 has been proposed as a new strategy to develop *PD-L1* inhibitors (95).

Drugs targeting other immune molecules are currently being investigated for treating MTC. V-domain Ig Suppressor of T-cell Activation (*VISTA*) membrane receptors are expressed not only by myeloid cells, but also by T cells. *VISTA* is expressed in tumor cells and regulates T cell function, thus promoting antitumor effects. In 2019, Marhelava et al. developed Onvatilimab for targeting the membrane receptor *VISTA* (96).

## Conclusion

**Figure 7:**
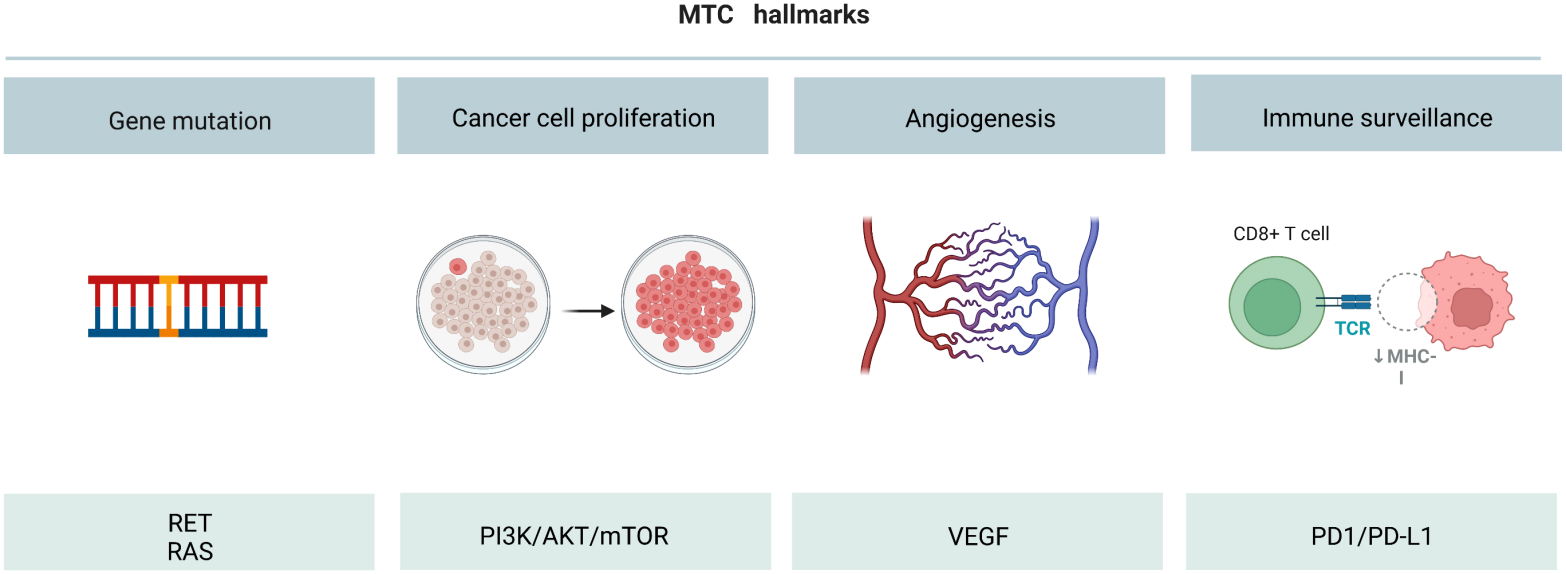
Overall diagram of MTC initiation, cell survival increase, angiogenesis and immune surveillance.

Although the 10-year survival rate of early stage MTC patients has reached more than 70%, the survival rate of late stage patients has reduced to less than 40% (4, 97, 98). Although several targeted drugs have been developed for the clinical treatment of MTC patients, their efficacy is still unsatisfactory. Therefore, it is urgent to further optimize the drug development strategy. Gene targeted therapy is the most effective method to treat tumors at present. For example, *RET*, *RAS*, *PI3Kk/AKT/mTOR*, *VEGF* and *PD-L1* play major roles in promoting MTC tumorigenesis (**Figure 7**). Development of targeted drugs has emerged as a hot spot in recent years. Increasing number of studies have identified the fusion or mutation sites of *RET* and *RAS* as potential targets for drugs. However, targeted inhibitors for these sites are yet to be developed. In the future, in addition to exploring and designing inhibitors of these new fusion and mutation sites, it is also necessary to develop small molecule inhibitors that can target and inhibit other signal transduction pathways, such as *RET-PTC1* and *RAS-PI3K*. Moreover, the *PI3K/AKT/mTOR* pathway is a classic signaling pathway that promotes tumorigenesis. The *mTOR* inhibitor everolimus, has been approved for clinical use for more than a decade, but drug resistance has always been a problem, and dual-target inhibitor has emerged as a potential solution. Moreover, classical TKIs inhibit cancer metastasis by introducing basic side chains at different positions of the TK and by binding to the hydrogen bonds. Moreover, VOA was shown to have an inhibitory effect on cancer cells through the formation of hydrogen bonds between the oxygen atom in the indole core of VOA and asn923 and cys919. The idea of directly combining the active residues of *VEGFR2* kinase domain to develop new drugs may also be explored in the future. In addition, the design and development of traditional immunotherapeutic drugs follow the concept of suppressing ADCC. Immunotherapy agents have shown remarkable efficacy both in animal studies and in the clinic. It is a promising strategy to synthesize new immune drugs that enhance the antitumor activity of MTC by adjusting the sliding docking fraction of PD-L1 and diphenyl ether or amine. All the above findings have provided new direction for future drug discovery for treating MTC.

## Data availability statement

The original contributions presented in the study are included in the article/supplementary material. Further inquiries can be directed to the corresponding authors.

## Author contributions

Conceptualization, YL and JQ. Methodology, HH. Software, XW. Validation, SZ, YL and JQ. Formal analysis, ZL. Investigation, KQ. Resources, SZ. Data curation, SZ. Writing—original draft preparation, YL. Writing—review and editing, JQ. Visualization, YL. Supervision, YL. Project administration, YL. Funding acquisition, YL. All authors contributed to the article and approved the submitted version.

## Funding

This research is funded by the Henan Science and Technology Key Project (222102310612).

## Acknowledgments

The authors would like to thank all the reviewers who participated in the review, as well as MJEditor (www.mjeditor.com) for providing English editing services during the preparation of this manuscript.

## Conflict of interest

The authors declare that the research was conducted in the absence of any commercial or financial relationships that could be construed as a potential conflict of interest.

## Publisher’s note

All claims expressed in this article are solely those of the authors and do not necessarily represent those of their affiliated organizations, or those of the publisher, the editors and the reviewers. Any product that may be evaluated in this article, or claim that may be made by its manufacturer, is not guaranteed or endorsed by the publisher.
